# Association of p62/SQSTM1 Excess and Oral Carcinogenesis

**DOI:** 10.1371/journal.pone.0074398

**Published:** 2013-09-24

**Authors:** Takuma Inui, Tokuhiro Chano, Mikiko Takikita-Suzuki, Masanori Nishikawa, Gaku Yamamoto, Hidetoshi Okabe

**Affiliations:** 1 Department of Clinical Laboratory Medicine, Shiga University of Medical Science, Otsu, Shiga, Japan; 2 Department of Oral and Maxillofacial Surgery, Shiga University of Medical Science, Otsu, Shiga, Japan; Virginia Commonwealth University, United States of America

## Abstract

p62/SQSTM1 (sequestosome1) has never been evaluated in oral epithelium. In order to clarify the role of p62/SQSTM1 in carcinogenesis in oral epithelium, both p62/SQSTM1 and Nrf2 were immunohistochemically evaluated in 54 carcinomas and 14 low grade dysplasias. p62/SQSTM1 knockdowns were also designed in oral cancer cells, and we analyzed the Nrf2 pathway, GSH contents and ROS accumulation. The association between p62/SQSTM1 excess and prognosis was addressed in a clinical cohort of oral carcinoma cases. p62/SQSTM1 excess was more obvious in carcinomas, but Nrf2 was abundant in almost all samples of the oral epithelium. In oral carcinoma cells, p62/SQSTM1 knockdown did not affect the Nrf2-Keap1 pathway but did significantly reduce GSH content with subsequent ROS accumulation, and caused cell growth inhibition in the irradiated condition. Finally, p62/SQSTM1 excess was associated with poor prognosis in a clinical cohort. In oral epithelial carcinogenesis, p62/SQSTM1 excess played a role in GSH induction rather than Nrf2 accumulation, and may cause resistance to cytotoxic stresses such as radiation or chemotherapy. Immunohistochemical evaluation of p62/SQSTM1 may be a potential significant marker to identify early carcinogenesis, chemo-radiotherapeutic resistance or poor prognosis of oral squamous cell carcinomas.

## Introduction

p62/SQSTM1 (sequestosome1) was isolated by cDNA library screening with autoantibody from patients with hepatocellular carcinoma (HCC) [1]. p62/SQSTM1 is stained in some carcinomas such as HCC [2], [3], intestinal carcinomas [4], anal carcinoma [5], and prostate carcinoma [6]. However, p62/SQSTM1 expression has never been evaluated in oral carcinomas and epithelia, and the contributed functions have never been analyzed in oral epithelial carcinogenesis.

Head and neck squamous cell carcinoma, such as oral carcinoma, is one of the carcinomas that are maximally associated with oxidative stress, and continuous oxidative stress can promote oncogenesis. Head and neck epithelium is often exposed to tobacco and alcohol, both sources of massive quantities of reactive oxygen species (ROS), which have been clearly identified as etiologic factors in these malignancies [7], [8]. Environmental and endogenous oxidative/electrophilic agents induce nuclear factor E2-related factor 2 (Nrf2), which is a master transcriptional activator of genes encoding numerous cytoprotective enzymes [9]. Accumulative Nrf2 mutations have been frequently observed in head and neck cancers, and Nrf2 is abundantly expressed in normal squamous epithelium in the regions. Therefore, aberrations of the Nrf2 pathway might play an important role in tissues exposed to abundant ROS, such as oral, nasopharyngeal, and tracheal epithelium [10]. Accumulative Nrf2 is currently recognized as one of the main cellular defense mechanisms against oxidative and electrophilic stresses [11]–[13]. Under quiescent conditions, the transcription factor Nrf2 is constitutively degraded through the ubiquitin-proteasomal pathway because its binding partner, kelch-like ECH-associated protein 1 (Keap1) is an adaptor of the ubiquitin ligase complex [14]–[17]. Exposure to electrophiles, ROS or nitric oxide instigates modification of the cysteine residues of Keap1, leading to its inactivation [18]–[20]. As a result, Nrf2 becomes stabilized and translocates to the nucleus to induce the transcription of numerous cytoprotective genes, including NAD(P)H dehydrogenase quinone 1 (NQO1), haem oxygenase-1 (HO-1) and glutathione *S*-transferase (GST) [21]–[24]. Excessive p62/SQSTM1 accumulates and binds to Keap1 in competition with Nrf2. Then, abundant, stabilized Nrf2 can induce the associated antioxidant proteins (NQO1 and HO-1) [25].

In the present study, p62/SQSTM1 excess was demonstrated in the oncogenic progression of oral squamous cell carcinoma, but Nrf2 was expressed in almost all samples of the oral epithelium. In order to analyze the roles of p62/SQSTM1 and the Nrf2-Keap1 pathway in oral epithelial carcinogenesis, oral cancer cells and p62/SQSTM1 knockdown variants were utilized. In this study we discovered an association between p62/SQSTM1 excess and poor clinical survival, and we propose that p62/SQSTM1 may be a potential novel predictive biomarker in the chemo-radiotherapeutic management of oral carcinoma cases.

## Materials and Methods

### Tissue samples and pathological data

Fifty-four primary oral squamous cell carcinomas and fourteen low grade dysplasias were obtained by biopsy at Shiga University of Medical Science. Patients with carcinoma comprised 32 males and 22 females with a median age of 64.8 years (27–92 years). The carcinoma specimens were removed from tongue (22 cases), gum (17 cases), buccal mucosa (4 cases), floor of mouth (4 cases), plate (2 cases) and lip (2 cases) tissues. Tumor classifications consisted of 23 cases of T1, 24 cases of T2, 2 cases of T3 and 5 cases of T4 (Table 1). Patients with low grade dysplasia comprised 8 males and 6 females with a median age of 56.9 years (29–82 years). The low grade dysplasias were removed from tongue (7 cases), gum (5 cases), buccal mucosa (1 case) and plate (1 case) tissues (Table 2). These cases were independent from carcinoma patients. Twenty-nine specimens of non-atypical epithelium consisted of lesions in separate regions of the specimens of cancers and dysplasias. Data were collected from clinical and pathological records with the written informed consent of individual patients and after approval by the Ethics Committee of Shiga University of Medical Science (Ethical Proof Number #22-105).

**Table 1:** Clinico-pathological parameters of the patients with oral squamous cell carcinomas.

Characteristics									No	( %)
Cases								54		
Average age at diagnosis								64.76		
Gender										
Male								32		(59.3)
Female								22		(40.7)
Tumor classification (T-stage)										
1								23		(42.6)
2								24		(44.5)
3								2		(3.7)
4								5		(9.3)
Histological differentiation										
Well differentiated								43		(79.6)
Moderately differentiated								8		(14.8)
Poorly differentiated								3		(5.6)
Location										
Tongue								22		(40.7)
Gum								17		(31.5)
Buccal mucosa								7		(13.0)
Floor of mouth								4		(7.4)
Palate								2		(3.7)
Lip								2		(3.7)

**Table 2:** Clinical parameters of the patients with low grade dysplasias.

Characteristics									No	(%)
Cases									14	
Average age at diagnosis									56.86	
Gender										
Male									8	(57.2)
Female									6	(42.9)
Location										
Tongue									7	(50)
Gum									5	(35.7)
Buccal mucosa									1	(7.2)
Palate									1	(7.2)

### Antibodies

Anti-p62/SQSTM1 (5F2) was obtained from MBL (Aichi, Japan). Anti-Nrf2 for immunohistochemistry (ab31163), anti-NQO1 (ab28947), anti-HO-1 (ab13248) and anti-Glutathione (gamma-glutamyl-cysteinyl-glycine, GSH; ab19534 [D8]) were obtained from Abcam (MA, US). Anti-phospho Nrf2 (pS40: 2073-1) for Western blot, which recognized the active nuclear form of Nrf2, was obtained from EPITOMICS (CA, US). Anti-Keap1 (10503-2-AP) was obtained from PROTEINTECH (IL, US). Anti-α-tubulin (DM 1A) was obtained from SIGMA (MO, US).

### Immunohistochemistry

Surgical specimens were transferred to 10% buffered formalin and fixed overnight. The fixed samples were embedded in paraffin, and serially sliced into 5µm sections. After dewaxing, sections were autoclaved at 120°C for 1min in 10 mM sodium citrate buffer (pH 6.0), and immersed in 0.3% H_2_O_2_. They were then incubated overnight at 4°C with primary antibodies to p62/SQSTM1 (diluted 1:500) or Nrf2 (diluted 1:200). The sections were rinsed with 1×PBS and incubated with the secondary antibody conjugated with horseradish peroxidase (Simple Stain MAX-PO; Nichirei, Tokyo, Japan) at room temperature for 1 hour. The sections were then stained with 3.3′-diaminobenzidinetetrahydrochloride (DAB) and counter-stained with hematoxylin. Xenografted human squamous cell carcinoma was treated with anti-cancer agents [26], and used for the positive control specimen. Non-immunized mouse or rabbit IgG was substituted for each primary antibody to serve as a negative control specimen. The staining was evaluated on a case-by-case basis by two blinded independent observers. A positive expression of p62/SQSTM1 or Nrf2 was defined when the cytoplasm and/or nuclei were stained with DAB. The staining grades were classified by the percentage of stained cells as follows: (–), the positive cells comprised <10%; (±), 10–20%; (+), >20%; (++), additional strong staining such as aggregation and nuclear accumulation, etc. In addition, ++/+ and ±/– were categorized as high and low expressions, respectively (Table 3). In 29 cases of non-atypical epithelium, the staining grades of non-atypical epithelium were compared with those of carcinomas or low grade dysplasias.

**Table 3:** Summary of the classical immunohistochemical staining scores.

p62/SQSTM1 and Nrf2		
High	++	>20% with aggregation or nuclear accumulation
	+	>20%
Low	±	10–20%
	-	<10%

### Proximity ligation assay

Proximity ligation assay (PLA) [27] was performed with Duolink II PLA probes and Detection Reagents Brightfield kit (Olink Bioscience, Uppsala, Sweden), following the manufacturer’s instructions. Tissues were deparaffinized, autoclaved, immersed in 0.3% H_2_O_2_ and incubated with overnight at 4°C with primary antibodies to p62/SQSTM1 (diluted 1:2000), Nrf2 (diluted 1:1000) or GSH (diluted 1:6000). PLUS and MINUS secondary PLA probes against mouse, rabbit or mouse IgG, respectively, were applied sequentially followed by hybridization, ligation, amplification and HRP/NovaRed detection solution. Image analysis was conducted as described previously [28]. Representative pathological regions of each case were reviewed by two pathology specialists (M.T. and H.O.), and 3–4 typical regions of each case were digitally photo-imaged. PLA signals of every image were counted semi-automatically (Figure S1) using the freely distributed software BlobFinder, which has been developed by the Centre for Image Analysis at Uppsala University (http://www. cb.uu.se/∼amin/BlobFinder/). Mean PLA signals from 3–4 images of each case were recognized as the case-originated values (RCPs/cell).

### Cell cultures

The cancer cell line SAS (human oral carcinoma) was purchased from Riken Cell Bank (Ibaraki, Japan). HeLa (human endocervical carcinoma) and CAL27 (human oral carcinoma) were from American Type Culture Collection. TIG-108 and –121 (human normal fibroblasts) were from the Japanese Collection of Research Bioresources. These cell lines were cultured in Dulbecco's modified Eagle's medium containing 10% fetal bovine serum (DMEM/10%FBS). Cell culture media were supplemented with penicillin (50 units/mL) and streptomycin (50 mg/mL). These cell lines were incubated at 37°C in a humidified chamber supplemented with 5% CO_2_.

### X-ray irradiation

For an X-ray irradiation treatment, subconfluent oral cancer cells of SAS and CAL27 in 6 well plates or 96 well plates containing DMEM/10%FBS were exposed to X-rays (2Gy/min) using a 150-kVp X-ray generator (Model MBR-1520R, Hitachi, Tokyo, Japan) with a total filtration of 0.5 mm aluminum filter, plus a 0.1 mm copper filter, and then incubated at 37°C in a conventional humidified 5% CO_2_ incubator.

### Protein extractions and Western blot analysis

The expressed proteins of each cell line were analyzed by immunoblotting. Cells were ruptured in ice-cold lysis buffer (40 mM HEPES (pH 7.5), 120 mM NaCl, 1 mM EDTA, 10 mM pyrophosphate, 10 mM glycerophosphate, 50 mM NaF, 0.5 mM orthovanadate, and EDTA-free protease inhibitors; Roche) containing 1% Triton X-100. Cell extracts were kept on ice for 20 minutes and centrifuged at 12,000 rpm for 10 minutes (4°C). The supernatants were boiled with SDS sample buffer. Proteins separated by SDS-PAGE were transferred to polyvinylidene difluoride filters. After blocking of the filters with TBS-T (10 mM Tris-HCl (pH 7.6), 150 mM NaCl, 0.1% Tween 20) containing 5% bovine serum albumin (BSA), the filters were incubated for 1 hour with the primary antibodies in TBS-T containing 2% BSA. The filters were then washed in TBS-T and incubated for 30 minutes in horseradish peroxidase-conjugated anti-mouse or anti-rabbit immunoglobulin G (GE Healthcare, US) diluted 1∶10,000 in TBS-T containing 2% BSA. After several washes with TBS-T, immunoreactivity was detected by the ECL system (GE Healthcare, US) according to the procedures recommended by the manufacturer.

### Preparation of recombinant lentiviruses encoding p62/SQSTM1 shRNA

Vectors for sh-RNA of p62/SQSTM1 (sh-p62(1) and (2)) and of nonsilencing control (sh-control) were purchased from OpenBiosystems (CA, US). Mature antisense sequences for p62/SQSTM1 were ATCAACTTCAATGCCCAGAGG and TTCTCTTTAATGTAGATTCGG, respectively (available at Thermo Scientific Bio, Web site). Lenti-virus for transferring each sh-RNA was prepared with each packaging mixture, according to the manufacturer’s instruction. SAS and CAL27 cells transferred by >10 MOI of each lenti-virus were selected in the presence of 3 µg/ml puromycin, expanded and used in the experiments.

### WST-8 assay for cell viability

SAS and CAL27 cells were cultured in 96-well plates at a density of 1×10^3^ per well, and cell viability assays were carried out by a Cell Counting Kit-8 (Dojindo Molecular Technologies Inc, Kumamoto, Japan). WST-8 reagent solution was added to each well, and the absorbance at 450 nm (OD450) was then measured by a microplate reader after the microplate was incubated with the reagent for 3 hours at 37°C, in accordance with the manufacturer's instructions.

### GSH and GSSG assay

Glutathione (GSH) and Glutathione-S-S-Glutathione (GSSG) were determined with a quantification kit (Dojindo Molecular Technologies Inc) according to the manufacturer’s instructions. The variously treated cells were lysed by the addition of 80 µL of 10 mM HCl and by freezing and thawing twice. To the homogenate was added 20 µL of 5% salicylsalicylic acid (SSA), and the mixture was centrifuged at 8,000x*g* for 10 minutes. GSH and GSSG levels in the supernatant were determined according to the manufacturer’s protocol by measuring absorbance at 405 nm with a microplate reader.

### ROS and DNA assays

ROS and DNA levels were detected with CellROX® Green reagent and Hoechst 33342 reagent (Life technologies, CA, US) containing excitation/emission at 485/520 nm and 352/461 nm, respectively. The cells were stained with 5 µM of CellROX® Green Reagent and 5 µg/ml of Hoechst 33342 reagent by adding the probe to the complete medium and incubating the cells at 37°C for 30 minutes. The cells were then washed with PBS, harvested by trypsin, immersed with Live cell imaging® reagents (Life technologies, CA, *US*), and analyzed on Attune® Acoustic Focusing Cytometer (Life technologies).

### Statistical analysis

To evaluate PLA signal differences statistically among non-atypical epithelium, low grade dysplasia and carcinoma; and to analyze assays for WST-8, GSSG and GSH among sh-RNA treated cells, one-way factorial ANOVA and multiple comparison tests accompanied by Scheffe's significance test were applied. To explore the clinical prognostic significance of p62/SQSTM1 excess, disease-specific survival curves were estimated by the Kaplan-Meier method with a log-rank test and the chi-square test. A *p*-value of <0.05 was considered statistically significant. All the statistical analyses were performed with StatView Version 5.0 for Windows (SAS institute Inc).

## Results

### p62/SQSTM1 excess was more obvious in oral squamous cell carcinomas than in low grade dysplasias or non-atypical epithelia

**Figure 1:**
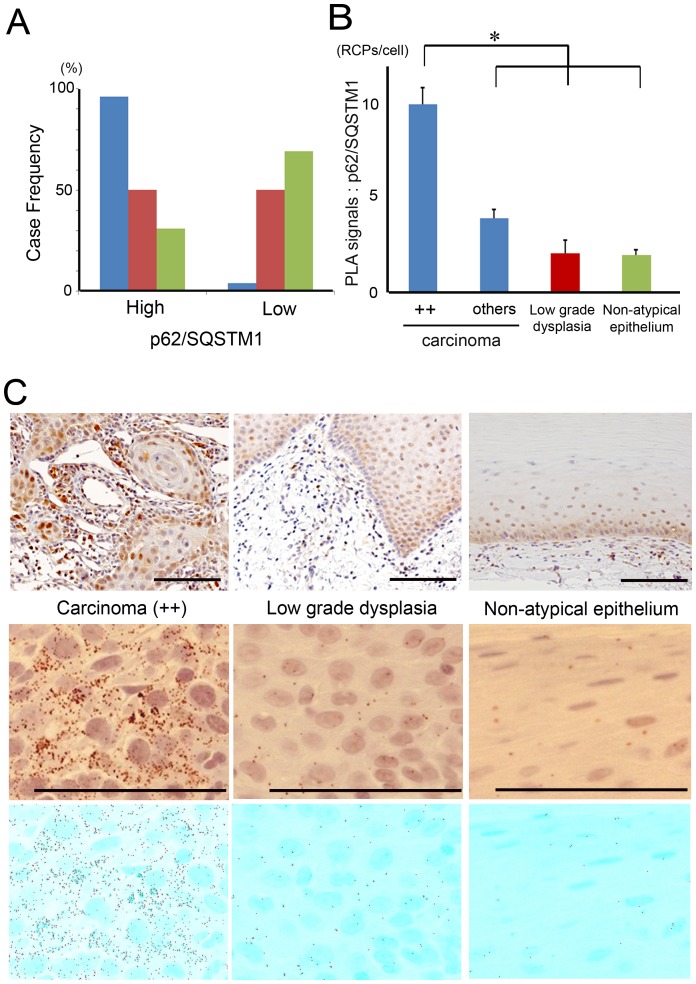
p62/SQSTM1 was abundantly stained in oral squamous cell carcinomas. (**A**) Case-frequencies (%) of p62/SQSTM1 staining grades in oral squamous cell carcinomas (blue columns; 54 cases), low grade dysplasias (red columns; 14 cases) and non-atypical epithelia (green columns; 29 cases). (**B**) Means ± S.E. of PLA signals for p62/SQSTM1 are displayed as bar graphs. The values (RCPs/cell) are 9.95±0.89, 3.90±0.48, 2.05±0.69 and 1.95±0.30 in the highest expression grade (++) carcinomas (24 cases), other carcinomas (30 cases), low grade dysplasias (14 cases) and non-atypical epithelia (29 cases), respectively. There was a significant difference between the highest expression grade (++) carcinomas and the other categories (*p*<0.0001), using one-way factorial ANOVA and multiple comparison tests accompanied by Scheffe's significance test. (**C**) Representative findings of p62/SQSTM1 staining in the highest expression grade (++) carcinoma (left), in low grade dysplasia (middle), and in non-atypical epithelium (right). Corresponding PLA signals and BlobFinder images are displayed in the middle and lower rows, respectively. Scale bar; 100 µm.

Clinical characteristics of the patients are summarized in Tables 1 and 2. The present cohort represented a population that was similar to that in the previous study [29]. In order to analyze the contribution of p62/SQSTM1 to carcinogenesis in oral carcinoma cases, expressional evaluation of p62/SQSTM1 was performed immunohistochemically. The case-frequency of immunohistochemical p62/SQSTM1 grades is summarized in a column graph. High-expression cases of p62/SQSTM1 were more frequently seen in oral squamous cell carcinomas than in low grade dysplasias or non-atypical epithelia. More than half of the cases in low grade dysplasias and in non-atypical epithelia were categorized as low expression. In contrast, only two cases (3.7%) were labeled as low expression in carcinomas (Figure 1A). In each specimen, PLA signals for p62/SQSTM1 were significantly increased in the carcinomas, especially in the highest expression grade (++) lesions (One-way factorial ANOVA and multiple comparison tests accompanied by Sheffe's significance test, *p*<0.0001; Figures 1B and 1C). Taken together, the results indicated that excessive p62/SQSTM1 was more significantly prominent in oral squamous cell carcinomas than in low grade dysplasias or non-atypical epithelia.

### Nrf2 was abundantly expressed in all the carcinomas, low grade dysplasias and non-atypical epithelia of oral tissues

**Figure 2:**
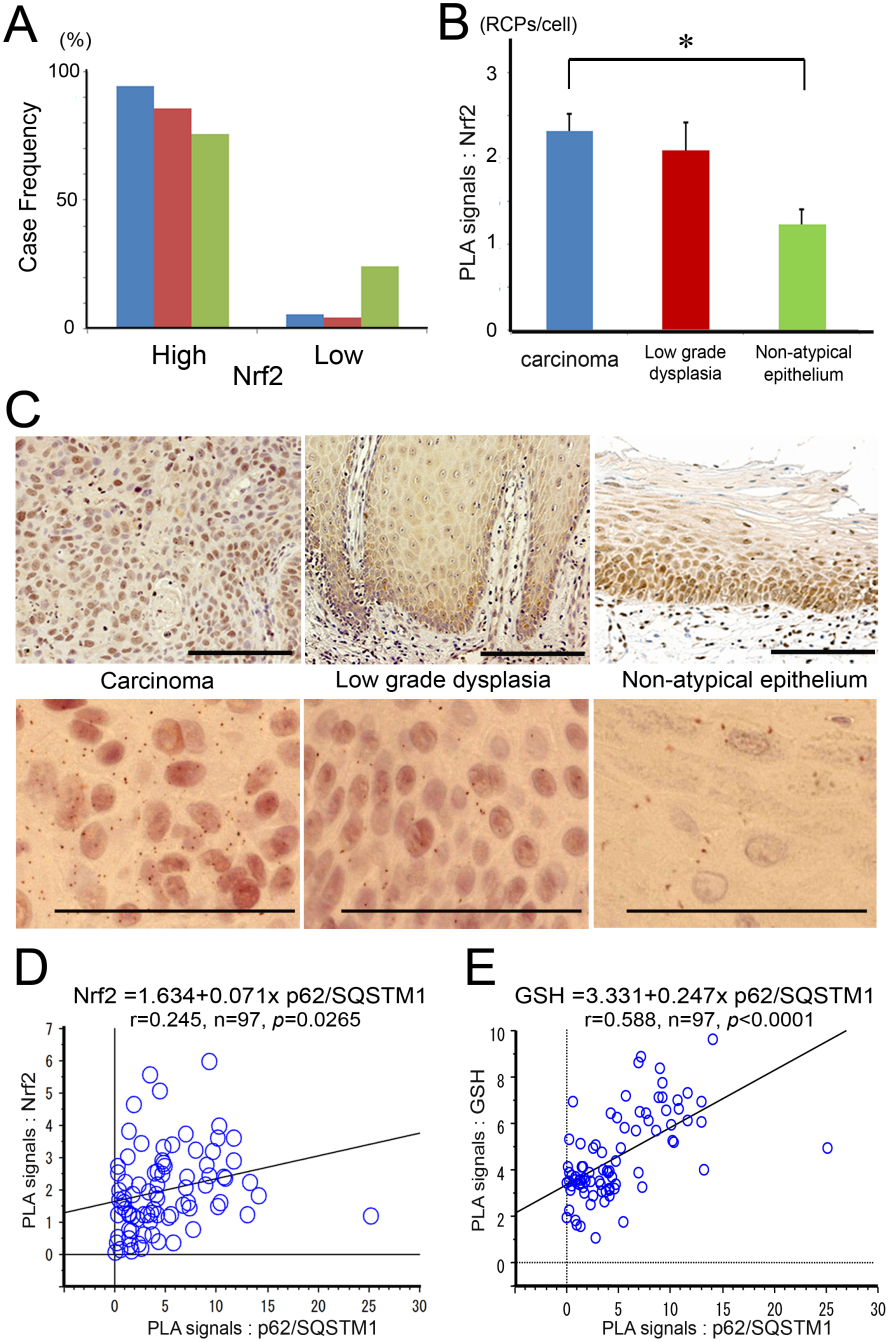
Nrf2 was abundantly expressed in carcinomas, low grade dysplasias, and non-atypical epithelia of oral tissue. (**A**) Case-frequencies (%) of Nrf2 staining grades in oral squamous cell carcinomas (blue columns; 54 cases), low grade dysplasias (red columns; 14 cases) and non-atypical epithelia (green columns; 29 cases). (**B**) Means ± S.E. of PLA signals for Nrf2 are displayed as bar graphs. The values (RCPs/cell) are 2.32±0.20, 2.10±0.32 and 1.24±0.17 in carcinomas (54 cases), low grade dysplasias (14 cases) and non-atypical epithelia (29 cases), respectively. There was a significant difference between carcinomas and non-atypical epithelia (*p* = 0.0029), using one-way factorial ANOVA and multiple comparison tests accompanied by Scheffe's significance test. (**C**) Representative findings of Nrf2 staining in carcinoma (left), in low grade dysplasia (middle), and in non-atypical epithelium (right). Corresponding PLA signals are displayed in the lower row. Scale bar; 100 µm. (**D**) There was a weakly positive correlation between p62/SQSTM1- and Nrf2- PLA signals (Pearson’s correlation coefficient; r = 0.245, n = 97, *p* = 0.0265). (**E**) There was a strongly positive correlation between p62/SQSTM1- and GSH-PLA signals (Pearson’s correlation coefficient; r = 0.588, n = 97, *p*<0.0001).

We examined Nrf2 expression immunohistochemically in order to evaluate its contribution to oral epithelial carcinogenesis. Nrf2 was abundantly expressed in all the samples of oral epithelia, ranging from normal epithelium to carcinoma. Almost all cases were categorized as high expression, and negative cases were never detected in any of the oral epithelia samples (Figure 2A). Quantified PLA signals for Nrf2 were significantly higher in carcinomas than in non-atypical epithelia (*p* = 0.0029), but not higher than that in low grade dysplasias (*p* = 0.8313, one-way factorial ANOVA and multiple comparison tests accompanied by Scheffe's significance test; Figures 2B and 2C). There was a weakly positive correlation between p62/SQSTM1- and Nrf2- PLA signals (Pearson’s correlation coefficient, r = 0.245, n = 97, *p* = 0.0265; Figure 2D). Glutathione (GSH) levels were additionally quantified by PLA in the clinical samples of oral epithelium, and then a stronger positive correlation between p62/SQSTM1- and GSH- PLA signals was significantly detected (Pearson’s correlation coefficient, r = 0.588, n = 97, *p*<0.0001; Figure 2E).

### p62/SQSTM1 knockdowns had little effect on Nrf2-Keap1 pathway in oral cancer cells

**Figure 3:**
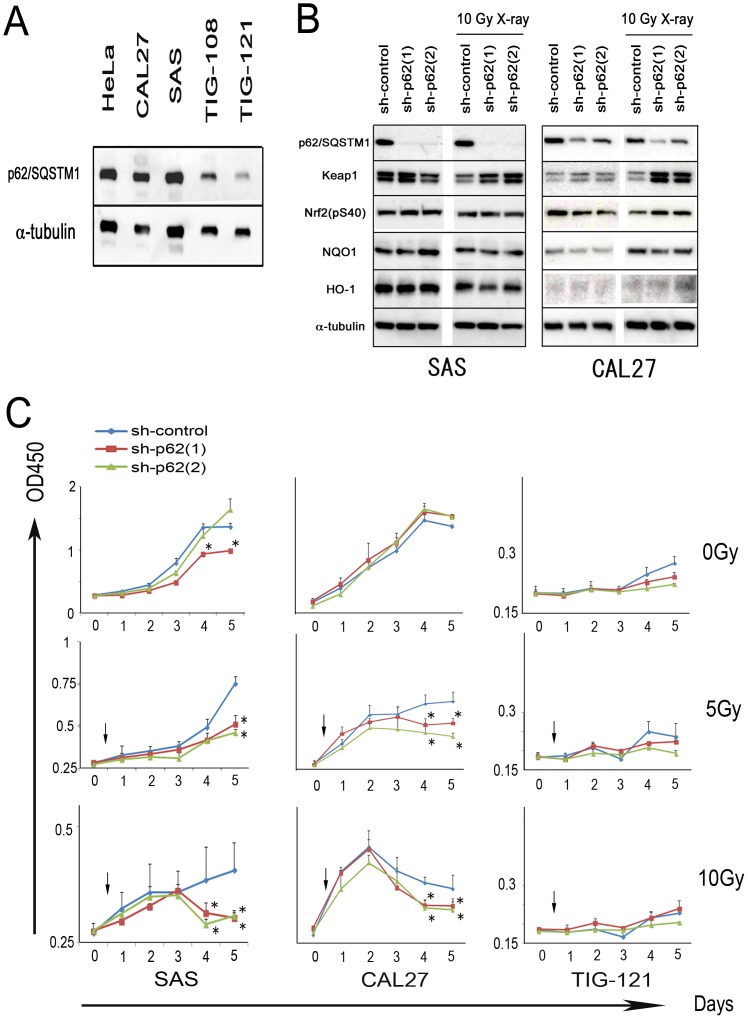
p62/SQSTM1 knockdown had little effect on the Nrf2-NQO1 pathway in oral cancer cells. However, p62/SQSTM1 knockdown affected the growth of the cells. (A) p62/SQSTM1 was abundantly expressed in SAS and CAL27 oral cancer cells. HeLa, endocervical carcinoma cells; TIG-108 and 121, normal human fibroblasts. (B) p62/SQSTM1 knockdown was performed by two kinds of shRNAs (sh-p62(1) and sh-p62(2)). shRNA for luciferase was used as control knockdown (sh-control). p62/SQSTM1 expression was significantly decreased by sh-p62(1) and sh-p62(2). Under no irradiation or 10 Gy X-ray irradiation, expressions of Nrf2 (pS40), Keap1, NQO1 and HO-1 were subtly affected by p62/SQSTM1 knockdown. Similar amounts of each cell protein were loaded in each lane of the SDS-PAGE. α-tubulin was used a loading control. Left and right panels indicate the data on SAS and CAL27, respectively. Three independent experiments were repeated, and the blotting photographs are representative ones. (C) p62/SQSTM1 knockdowns and X-ray irradiation were similarly performed. No radiation (upper); The effects of shRNA (sh-p62(1) and sh-p62(2)) were partial under normal culture condition. X-ray irradiations of 5 Gy (middle) and 10 Gy (lower); The cancer cell growth was significantly inhibited by two kinds of shRNA for p62/SQSTM1. Left and middle panels indicate the data on SAS and CAL27, respectively. The data on TIG-121 are displayed as a reference at the right panels. In the WST-8 assays, mean absorbance values (OD450) ± SE are shown vertically, and the number of days after exposure to radiation is indicated horizontally. The values are derived from quadruplicate experiments (*, *p*<0.05, one-way factorial ANOVA and multiple comparison tests accompanied by Scheffe's significance test).

p62/SQSTM1 was highly expressed in oral cancer cells of SAS and CAL27 compared with expression in human fibroblasts (TIG-108 and -121; Figure 3A). Excessive p62/SQSTM1 binds to Keap1 competitively with Nrf2, and can cause induction of the antioxidant proteins (NQO1 and HO-1) [25]. Therefore, to explore p62/SQSTM1 contribution to the Nrf2-Keap1 pathway, knockdowns were designed in two kinds of oral cancer cells, SAS and CAL27. p62/SQSTM1 was significantly depressed by the shRNA (sh-p62 (1) and sh-p62 (2)). Keap1 was abundantly detected by sh-RNAs for p62/SQSTM1 under 10 Gy X-ray irradiation, although the expression was not significantly changed under normal conditions. However, expressions of active Nrf2, NQO1 and HO-1 were subtly affected by p62/SQSTM1 knockdown, and no significant constant effect was detected under normal or 10 Gy X-ray irradiated conditions (Figure 3B). The data suggested that the role of p62/SQSTM1 could not be adequately explained in the cascade from Nrf2-Keap1 to NQO1 and HO-1.

### Under irradiation, cancer cell growth was significantly inhibited by p62/SQSTM1 knockdown in oral cancer cells

The growth of oral cancer cells treated with shRNA (sh-control, sh-p62 (1) and sh-p62 (2)) were evaluated under conditions with/without X-ray irradiation. Under 5 and 10 Gy X-ray irradiation, shRNAs for p62/SQSTM1 (sh-p62(1) and sh-62(2)) caused significant growth inhibition, although the effects were partial under normal culture conditions (one-way factorial ANOVA and multiple comparison tests accompanied by Scheffe's significance test, *p*<0.05; Figure 3C). These data suggested that p62/SQSTM1 played a more important role in the survival of irradiation than in normal conditions.

### p62/SQSTM1 knockdowns significantly reduced GSH content in oral cancer cells

**Figure 4:**
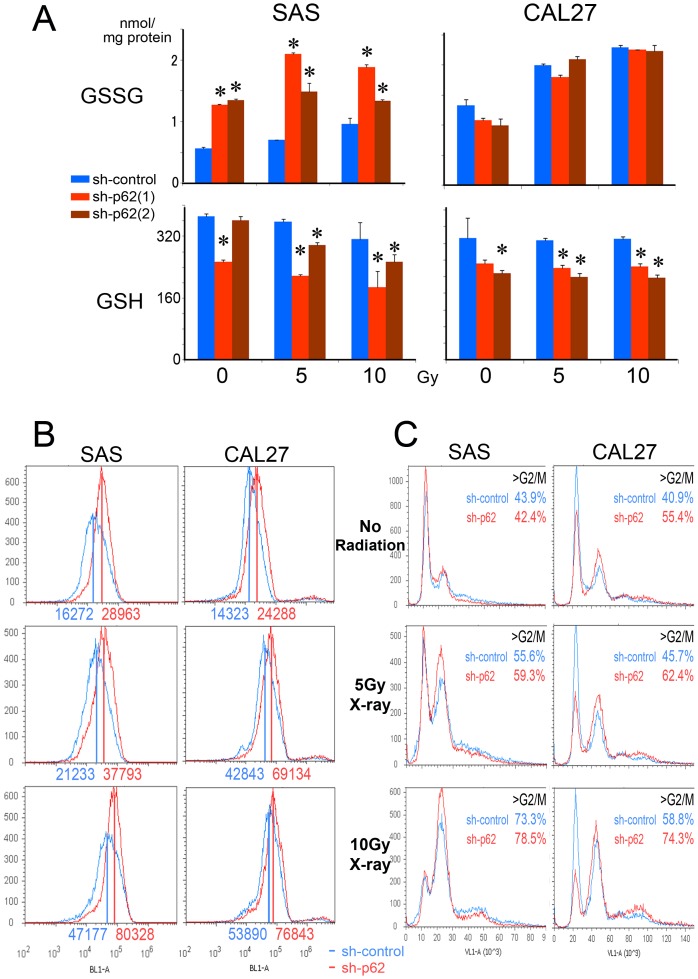
GSH cannot be induced by the irradiation, and ROS accumulate in p62/SQSTM1 knockdown cells. (**A**) GSSG (upper) and GSH (lower) levels of shRNAs (sh-control, sh-p62(1) and sh-p62(2); blue, red and brown columns, respectively) -treated cells are indicated by bar chart. In each graph, the left 3 columns indicate the data of no radiation. The middle 3 and right 3 columns indicate the data of 5-10 Gy X-ray irradiation followed by 24h culture. Although GSH was maintained in sh-control cells, it was significantly reduced in sh-p62(1) and sh-p62(2) cells. The values are derived from triplicate experiments (*, *p*<0.05, one-way factorial ANOVA and multiple comparison tests accompanied by Scheffe's significance test). (**B**) ROS levels were detected with CellROX® Green reagent in sh-control and sh-p62 cells, under no radiation (upper) and 5 (middle) - 10 (lower) Gy X-ray irradiation. ROS levels in p62/SQSTM1 knockdown cells (red lines) were higher than in control cells (blue lines). Numbers indicate mean fluorescent values. (**C**) DNA contents were evaluated with Hoechst 33342 reagent in sh-control and sh-p62 cells, under no radiation (upper) and 5 (middle) - 10 (lower) Gy X-ray irradiation. The more cells were irradiated, the more prominently >G2/M phase cell fractions were accumulated. The accumulation was more prominent in p62/SQSTM1 knockdown cells (sh-p62; red lines) than in the control cells (sh-control; blue lines). Sub-G1 apoptotic fractions could not be detected. Left and right panels indicate the data on SAS and CAL27 cells, respectively.

To evaluate whether p62/SQSTM1 was essential to induce a GSH response to X-ray irradiation, GSSG and GSH levels were analyzed in p62/SQSTM1-knockdown oral cancer cells. GSSG levels were increased in the knockdown cells of SAS (sh-p62(1) and sh-p62(2); red and brown columns; Figure 4A). Under no irradiation, GSH levels were mildly affected by p62/SQSTM1 knockdown in oral cancer cells (Figure 4A). GSH was significantly reduced under 5-10 Gy X-ray irradiation in both oral cancer cells treated with shRNAs for p62/SQSTM1 (sh-p62(1) and sh-p62(2); red and brown columns), although it was maintained under 5-10 Gy X-ray irradiation in the control shRNA cells (sh-control; blue columns; one-way factorial ANOVA and multiple comparison tests accompanied by Scheffe's significance test, *p*<0.05; Figure 4A). These data indicated that p62/SQSTM1 was significantly involved in GSH maintenace in oral cancer cells.

### ROS levels were significantly increased and catastrophic growth-inhibition was promoted by p62/SQSTM1 knockdown in oral cancer cells

In order to analyze whether more ROS was accumulated in p62/SQSTM1-knockdown cells, ROS levels were quantified in the knockdown and control variants of SAS and CAL27 oral cancer cells. ROS levels were higher in p62/SQSTM1-knockdown cells (sh-p62; red lines) than in the control oral cancer cells (sh-control; blue lines) under all the irradiated conditions (Figure 4B). The cells containing high amounts of ROS were prominently detected under 10 Gy X-ray irradiation in sh-p62 cells (Figure 4B). Sub-G1 apoptotic fractions could not be detected in SAS and CAL27 oral cancer cells. However, in cells that were more irradiated, accumulation of >G2/M phase cells became more prominent. The accumulation was more siginificant in p62/SQSTM1-knockdown cells (sh-p62; red lines) than in the control cells (sh-control; blue lines; Figure 4C). Both the prominent accumulation of >G2/M phase cells and the growth-inhibition suggested mitotic catastrophe, which is one type of cell death that may occur after irradiation. The data on two kinds of p62/SQSTM1 knockdown cells (sh-p62(1) and sh-p62(2)) were quite similar in all the conditions, so the data on sh-p62(1) cells are shown as representative examples.

### p62/SQSTM1 excess indicated an important clinical relevance in oral cancer

**Figure 5:**
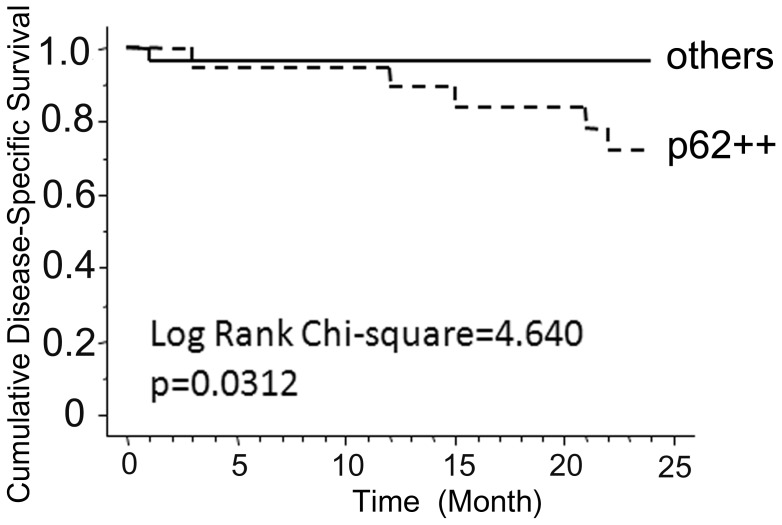
p62/SQSTM1 excess predicts clinically worse prognoses in oral cancer cases. Forty-nine cases of oral cancer were immunohistochemically evaluated for p62/SQSTM1 and statistically analyzed relative to the clinical outcomes. Kaplan-Meier curve with log-rank tests was performed for disease-specific survival in relation to p62/SQSTM1 level. (Chi-Square value = 4.640, p = 0.0312).

Finally, we evaluated whether p62/SQSTM1 excess was associated with poor prognosis in a clinical cohort. Forty-seven cases of oral cancer could be immunohistochemically evaluated and followed-up clinically. The association between p62/SQSTM1 staining and clinical disease-specific survival was evaluated statistically in the Kaplan-Meier curve together with log-rank analysis (Figure 5). The immunohistochemical status of p62/SQSTM1 could predict the clinical prognosis of oral cancer cases, and the cases with strong p62/SQSTM1 staining showed the worse prognoses with regard to a 2-year survival.

## Discussion

The present study has demonstrated for the first time that an abnormal excess of p62/SQSTM1 is present in oral squamous carcinoma cells. The p62/SQSTM1 excess was more obvious in oral carcinomas than in low-grade dysplasias or non-atypical epithelia. p62/SQSTM1 excess can cause low grade dysplasia in the oral epithelium to develop into carcinoma via the resistance to various oxidative stresses. In order to investigate the association between p62/SQSTM1 excess and the antioxidant system in oral squamous epithelium, Nrf2 expression was analyzed. Nrf2 was abundantly expressed in all the oral epithelial samples ranging from normal epithelium to carcinoma. Nrf2 produces antioxidant proteins, which provide resistance to the cytotoxicity caused by oxidative stress, ROS, and/or subsequent DNA damage [11]-[13]. Oral squamous epithelium is ordinarily exposed to oxidative stress from substances like tobacco and alcohol, and Nrf2 is constitutively expressed in such epithelium [7]. Nevertheless, the association of Nrf2 with oral epithelial carcinogenesis has not been adequately explained.

In order to precisely explore a p62/SQSTM1 contribution to the Nrf2 pathway in oral epithelial carcinogenesis, p62/SQSTM1 knockdowns were designed in oral cancer cells, where p62/SQSTM1 was highly expressed. It has been reported that p62/SQSTM1 accumulates and activates Nrf2 by competing with Nrf2 for its binding to Keap1 [25]. Under 10 Gy X-ray irradiation, Keap1 was abundant in p62/SQSTM1-knockdown cells, and it seems likely that p62/SQSTM1 insufficiency could reduce Keap1 degradation through a dysfunctional autophagic-lysozome pathway. However, the present study demonstrated that p62/SQSTM1 knockdowns did not largely affect active Nrf2 and the associated antioxidant proteins (NQO1 and HO-1) under normal culture conditions and 10 Gy X-ray irradiation. The data suggested, therefore, that p62/SQSTM1 does not contribute significantly to Nrf2 activity and the associated pathway in oral cancer cells.

The oral cancer cell growth was inhibited by p62/SQSTM1 knockdowns especially under X-ray irradiated conditions. This result suggested that p62/SQSTM1 may play an important role in oral cancer cell survival. GSH content is tightly regulated in the maintenance of the cellular redox status [30] and has been implicated in protection against environmental oxidant-mediated injuries [31]. Normally, in cells and tissues, more than 90% of the total glutathione pool is in the reduced form, GSH, and less than 10% exists in the disulfide form, GSSG. Changes in the ratios of both GSH and GSSG can broadly affect signaling pathways participating in physiological responses associated with cell proliferation, autophagy and apoptosis [31]. Therefore, the present study focused on understanding the association between GSSG-GSH and p62/SQSTM1 in X-ray irradiated oral cancer cells producing ROS.

p62/SQSTM1 knockdown significantly reduced GSH contents and increased ROS levels in oral cancer cells. Especially under X-ray irradiated conditions, GSH induction was never seen in the knockdown cells, although GSH levels were maintained in the control cells. These data suggested that in control cells GSH induction responded to X-ray irradiation in order to diminish the produced ROS, and that p62/SQSTM1 insufficiency resulted in failure to induce GSH and failure to protect the oral carcinoma cells from cytotoxic stress. In other words, p62/SQSTM1 excess cannot play a role in Nrf2 accumulation, but it instead helps to sustain GSH concentration in response to ROS in the oral cancer cells. Apoptotic cell death could not be detected under X-ray irradiated conditions in oral cancer cells, but the prominent accumulation of >G2/M phase cells was shown especially in p62/SQSTM1-knockdown cells, suggesting catastrophic growth-inhibition. Probably, insufficient p62/SQSTM1 causes GSH deficit, ROS accumulation, and the resultant growth-inhibition known as mitotic catastrophe.

It has been reported that p62/SQSTM1 regulates predominantly GSH production rather than Nrf2 accumulation in retinal pigment epithelial cells [32], where photo-oxidative stresses are abundant. Similarly, p62/SQSTM1 did not only correlate with Nrf2, but also contributed more prominently to increase GSH contents in the clinical samples of the present study. Therefore, in oral epithelial cells, Nrf2 proteins are maintained against ordinary oxidative stresses. In such oxidative environments, p62/SQSTM1 predominantly induces sufficient amounts of GSH to destroy various strong cytotoxic stresses caused by radiation or chemotherapy, and then contributes to oral epithelial carcinogenesis.

Finally, in order to clarify whether excessive p62/SQSTM1 could be associated with a clinically poor prognosis in oral cancer cases, the cohort study was performed. In fact, classical immunohistochemical evaluation of p62/SQSTM1 can provide a predictive benefit in the clinical management of oral cancer patients, and further analysis validating the clinical prognostic value will be warranted. It has been reported that there is an association between p62/SQSTM1 staining and clinical survival in lung cancers [33]. Similarly, the present study indicates a possible predictive value of p62/SQSTM1, and additionally suggests that excess p62/SQSTM1 appears to be correlated with the resistance to oxidant stresses caused by radiotherapeutic treatments.

p62/SQSTM1 excess may play a role in oral epithelial carcinogenesis through the corresponding GSH induction in response to cytotoxic stresses such as radiation or chemotherapy. Immunohistochemical evaluation of p62/SQSTM1 may prove to be a potentially significant test that will help to identify early carcinogenesis, chemo-radiotherapeutic resistance or poor prognosis of oral squamous cell carcinomas.

## Supporting Information

Figure S1 **PLA signals of each case were adequately counted.** Representative pathological regions of each case were reviewed by two pathology specialists (M.T. and H.O.), and 3-4 typical regions of each case were digitally photo-imaged (upper and middle rows). PLA signals of every image were counted semiautomatically using the software BlobFinder (lower row). The mean PLA signals from 3-4 images of each case were recognized as the case-originated values (RCPs/cell).
(TIF)
