# Supplementary figures and images for: Association of p62/SQSTM1 Excess and Oral Carcinogenesis
Source: PLoS One. 2013 Sep 24;8(9):e74398. doi: 10.1371/journal.pone.0074398 (PMC3782476; doi:10.1371/journal.pone.0074398)

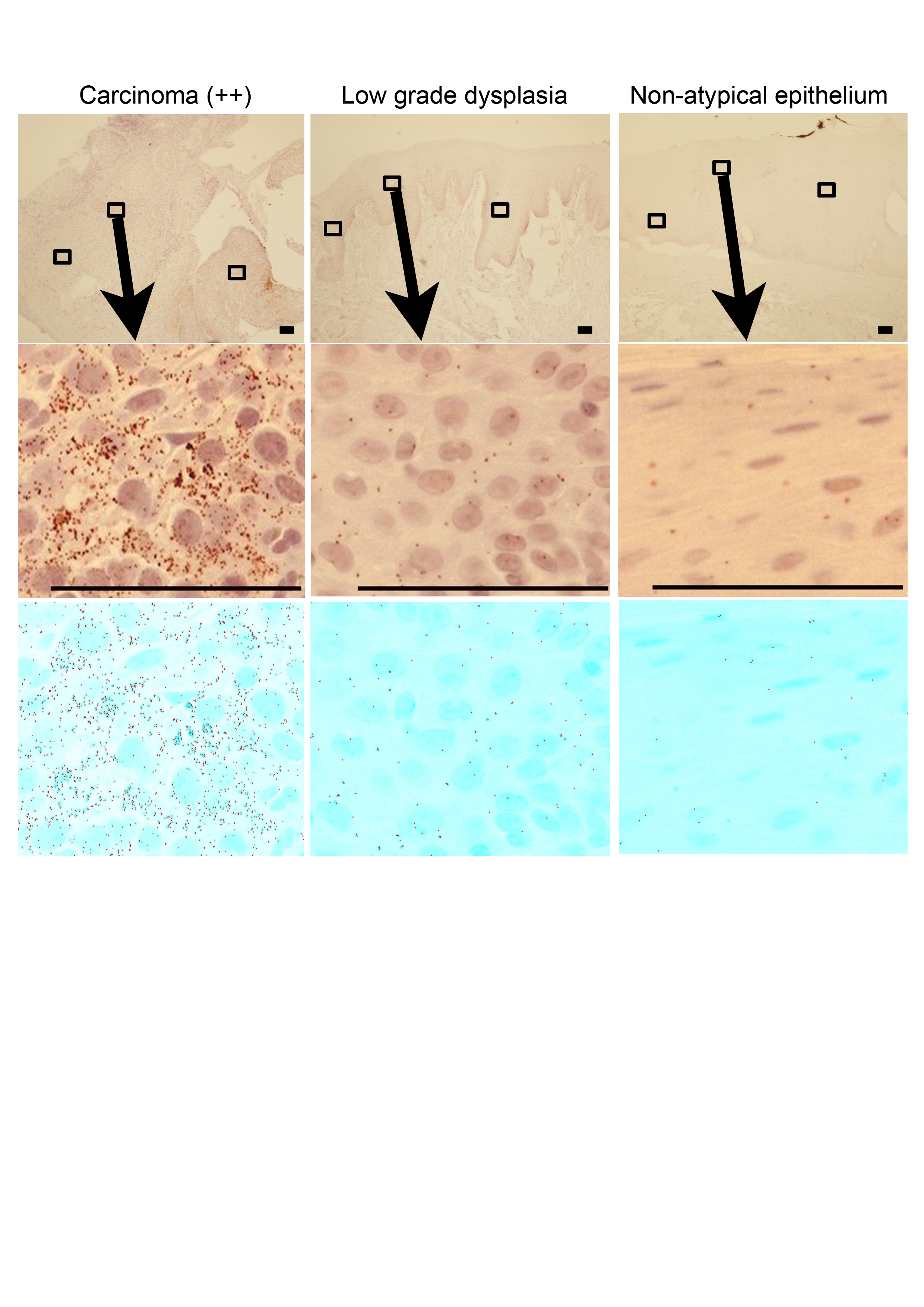

Supplement: Figure S1 — PLA signals of each case were adequately counted. Representative pathological regions of each case were reviewed by two pathology specialists (M.T. and H.O.), and 3-4 typical regions of each case were digitally photo-imaged (upper and middle rows). PLA signals of every image were counted semiautomatically using the software BlobFinder (lower row). The mean PLA signals from 3-4 images of each case were recognized as the case-originated values (RCPs/cell). (TIF) [file pone.0074398.s001.tif]
